# A spherical falling film gas-liquid equilibrator for rapid and continuous measurements of CO_2_ and other trace gases

**DOI:** 10.1371/journal.pone.0222303

**Published:** 2019-09-25

**Authors:** A. Whitman Miller, Amanda C. Reynolds, Mark S. Minton

**Affiliations:** Smithsonian Environmental Research Center, Edgewater, Maryland, United States of America; University of Akron, UNITED STATES

## Abstract

Use of gas-liquid equilibrators to measure trace gases such as CO_2_, methane, and radon in water bodies is widespread. Such measurements are critical for understanding a variety of water quality issues such as acidification due to elevated CO_2_ or other processes related ecosystem metabolism and function. However, because gas-liquid equilibrators rely on generating sufficient surface area for gas exchange between liquid and gas phases, most traditional equilibrators pass water through small orifices or interstitial spaces that rapidly clog in highly productive or turbid waters, conditions that are common in estuaries, coastal bays, and riverine systems. Likewise, in cold temperatures, such equilibrators are subject to freezing. Both situations lead to failure and limit utility, especially for long term, continuous environmental monitoring. Here we describe and test a gas-liquid equilibrator that relies on a continuous falling film of water over a spherical surface to drive gas exchange. Our results demonstrate that this design is accurate in its ability to equilibrate fully to aqueous CO_2_ concentrations, is functional across a wide range of gas concentrations, and has a response time that is comparable with other equilibrator designs. Because this equilibrator uses free flowing, falling water to produce a surface for gas exchange, our field trials have shown it to be very resistant to clogging and freezing, and therefore well suited to long term deployment in highly productive waters like estuaries where CO_2_ concentrations fluctuate hourly, daily, and seasonally. When generated across a spherical surface, the falling film is not adversely affected by tilting off vertical, conditions that are common on a ship, small vessel, or buoy.

## Introduction

Advancing our understanding of the pace of climate change in aquatic ecosystems requires specialized instruments that can monitor carbon dioxide and other trace gases in extremely variable environments such as highly productive, seasonally varying, or degraded environments with high suspended sediment loads. Gas-liquid equilibration has proven to be a useful approach for measuring trace gases in water, but most designs for equilibrators have important flaws that limit their effectiveness. Yoon et al. [1] review active, passive, discrete, and continuous methods for gas-liquid equilibration, including manual headspace, spray-type, marble-type, and submerged membrane gas-liquid equilibration techniques, and describe the relative merits and shortcomings of each for measurements across a broad range of pCO_2_ in inland waters. Many traditional equilibrators (EQL) are prone to biofouling and/or blockage of internal passages after hours or days of deployment in some coastal systems [2]. Turbidity, debris, and biological growth in warm months, and freezing temperatures in cold months, if not properly guarded against, can result in EQL failure and even flooding of the system, either of which yield faulty data.

As part of our investigations of water quality and ecosystem metabolism of the Chesapeake Bay and its tidal estuaries, we have monitored daily and seasonal variation in dissolved carbon dioxide in a variety of specific local environments, both at fixed monitoring locations and along underway transects. Estuaries and riverine ecosystems are important drivers of the local and regional biogeochemistry of coastal areas, and contribute significantly to the global carbon cycle [3–8]. These ecosystems can undergo rapid changes in CO_2_ due to biological activity (e.g., photosynthesis and benthic respiration, exacerbated by land-sea exchanges, and eutrophication), so frequency of measurement is vital for understanding temporal and spatial heterogeneity of these ecosystems [9–12]. We routinely measure volume mixing ratios (xCO_2_) in the range of < 100 ppmv to > 4,000 ppmv, but sometimes observe > 30,000 in the Chesapeake Bay, depending on location and time of year. Among the greatest challenges we have encountered is the reliability of gas-liquid equilibration devices (i.e., equilibrators or EQLs) to operate effectively during long term deployments in the face of extreme biofouling, turbidity, and freezing.

In this paper we describe and test a novel gas-liquid EQL design based on a falling film of water over a spherical surface. We investigate the behavioral characteristics of the spherical falling film EQL design to rapidly equilibrate a measurement gas (i.e., a closed loop of headspace air that circulates between EQL and sensor) with ambient carbon dioxide (CO_2_) concentrations in sample water that is continuously refreshed by pumping from the source water being measured. Although the present investigation focuses exclusively on the dynamics of measuring CO_2_, we believe that the simple but robust design of the falling film EQL makes it an excellent alternative for many applications, especially in environments that are turbid or have high rates of biofouling. Further, the spherical, or substantially spherical (e.g., spheroidal, ovoidal, ellipsoidal) EQL produces a stable and efficient gas exchange surface across a broad range of water flow rates, the function of which is undisturbed by physical jostling or tipping.

A variety of gas-liquid equilibration mechanisms have been used for making trace gas measurements and have been described by a number of investigators—see summaries and reviews by Yoon et al. [1] and Webb et al. [13]. Here, we focus on active pumping, continuous equilibration methodologies (i.e., automated, continuous gas-liquid equilibration).

### Gas-liquid equilibrators

Körtzinger et al. [14] identify and summarize the characteristics of three primary types of air-water EQLs: shower-, bubble-, and thin film-types. Frankignoulle et al. [9] introduced a marble-type EQL to address issues of high turbidity and large swings in pCO_2_ over space and time. We provide a brief review of these EQL types for comparison and to give context for our spherical falling film EQL design.

#### Shower-type equilibrators

The showerhead EQL forces water under pressure through a collection of small orifices of a nozzle to produce tiny water droplets that have extensive surface area for rapid gas exchange across the gas-liquid interface in the headspace of the EQL. The Weiss EQL introduces water through a collection of drill holes in the EQL chamber’s roof and gas exchange is generated as the water trickles through the EQL headspace [15]. The showerhead and Weiss EQLs produce efficient air-water equilibration and are likely the most widespread gas-liquid EQLs in use [1,13]. However, when phytoplankton density is high, or the water is especially turbid or debris-ridden, the nozzle of the showerhead and drilled holes of the Weiss EQL can rapidly clog, leading to malfunction. Clogging thus precludes extended field deployment of showerhead EQLs in many aquatic environments. Although water can be filtered upstream from the showerhead EQL, maintaining adequate filtration requires extensive maintenance and cleaning, again becoming impractical for deployments of more than a few hours in a muddy estuary or similarly turbid water body.

#### Thin film equilibrators

Rather than producing small droplets of water, thin film EQLs maximize wetted surface area of solid surfaces for rapid gas-liquid equilibration. Thin film EQLs rely on continuously renewed thin films of water to form the site for gas exchange across the air-water interface. Sabine and Key [16] designed, tested, and deployed a motorized rotating disc EQL that has been used extensively in underway ocean surveys. Poisson et al. [17] describe a thin-film EQL that uses falling water to create a thin water coating on the inner walls of a columnar chamber and a counter-current upward flow of air in the inner chamber to provide equilibrating conditions. Another variant of the thin film EQL described by Frankignoulle et al. [9] is the marble-type EQL which is a column filled with marbles that creates a packed bed-like exchange matrix with a high surface area to volume ratio to maximize gas exchange as water introduced to the top of the EQL flows through the marbles. The marble-type EQL designs have been used effectively in estuaries and streams across a broad range of pCO_2_ levels and have been shown to have fast response times due to their maximized surface area [1,9,10,13]. In our experience, although the marble-type is a vast improvement over the showerhead and Weiss EQLs in estuarine settings, it still becomes clogged and malfunctions within a few days of deployment in the highly productive Chesapeake Bay.

In our research and field monitoring, we have explored the use of thin film EQLs of various designs. One variant we have used extensively is a multi-chambered, thin film EQL based on the original single chamber design of Poisson et al. [17]. In each of the columnar chambers (38 mm diam), pressurized water is introduced horizontally near the top of each chamber, wetting the inner surface of each chamber wall, creating a thin film. This EQL design is an open loop system that uses atmospheric air as the carrier gas. Atmospheric air is introduced into the bottom of one chamber and this air is routed in serial and counter-current to the falling water in each of the chambers, progressively bringing the air into equilibrium with the water to a point where exiting air is effectively in equilibrium with the entering water (S1 Fig). This equilibrated air is dehumidified and passed through a non-dispersive infrared gas analyzer (IRGA) and exhausted to the atmosphere.

The multi-chamber thin film EQL is able to fully equilibrate water across a wide range of pCO_2_ conditions (i.e., sub-atmospheric to several thousand ppmv), and is far less prone to clogging than shower-type designs. This EQL can be deployed, unattended, for many days at a time, even in the highly productive waters of the Chesapeake. Some shortcomings of this design include the difficulty and frequency of cleaning, and the necessity that chambers remain plumb to the surface of the water for proper operation, limiting its capacity on floating platforms that may sway or rock the apparatus.

### Spherical falling film EQL

**Fig 1:**
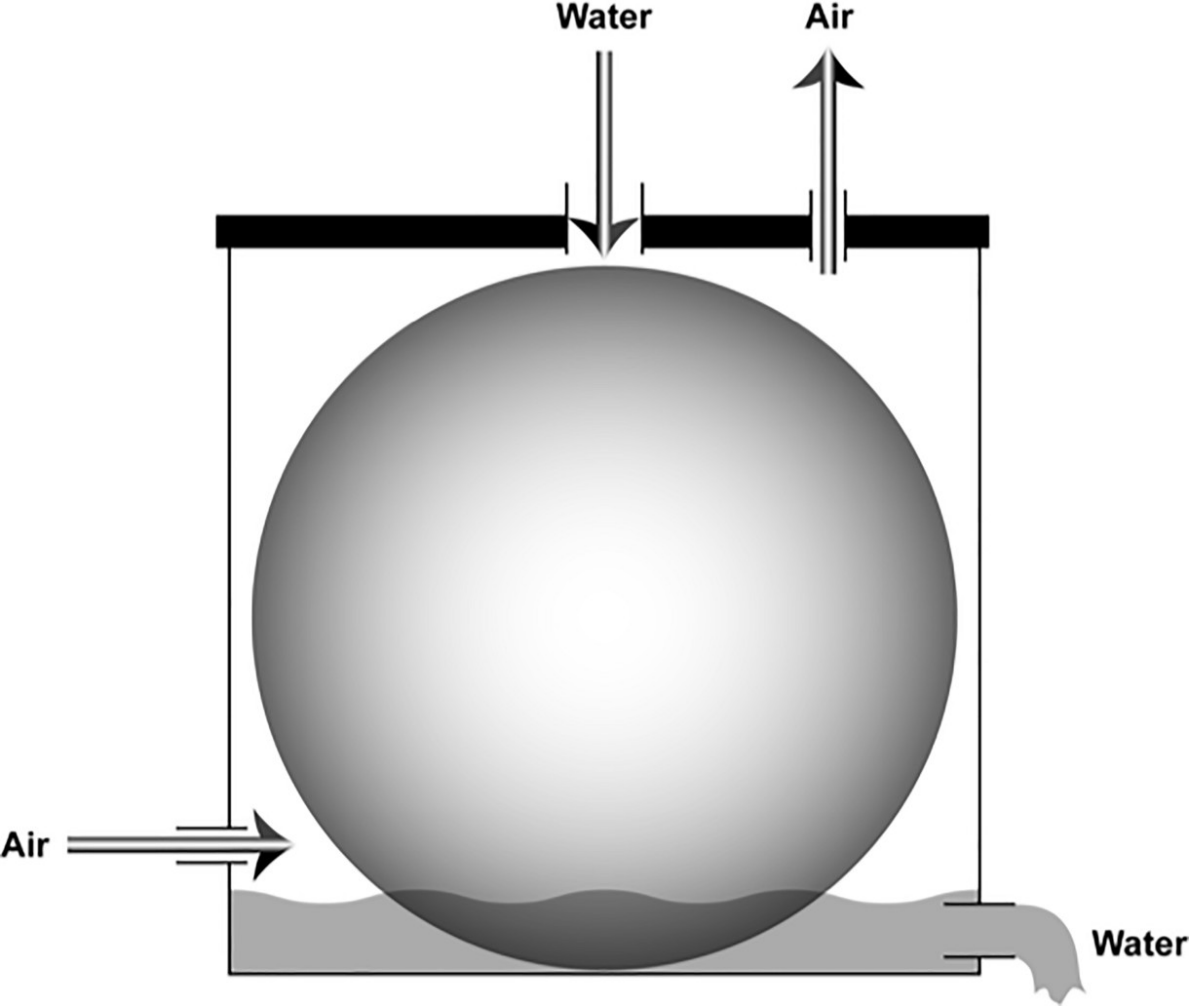
Spherical falling film equilibrator diagram. Water is pumped through the roof of the chamber and directly onto the topmost point of sphere. The water forms a falling film that coats the sphere and creates a gas exchange surface. Water releases from the sphere at the water line below, where it drains out of the chamber. Air enters the lower part of the chamber, flows upward, counter-current to the water flow, and exits out the roof of the chamber. Air is circulated in a closed loop from the EQL to a sensor and back into the bottom of the chamber.

To more fully address the challenges of making pCO_2_ measurements in highly turbid and productive waters, where the pCO_2_ can routinely vary from sub-atmospheric to tens of thousands of ppmv [1,5], we designed a falling film EQL with a spherical equilibration member that serves as the reaction surface (Fig 1). Traditionally falling film evaporators have been used in industrial settings for chemical and petroleum refining, refrigeration, desalinization, air conditioning, and in the food and dairy industry [18–20]. Because falling films have tremendous heat exchange potential, understanding the geometry and fluid dynamics that yield optimal heat exchange for use in industrial processes has been of great interest for decades [20]. Falling films are typically generated across the inner or outer walls of vertical pipes, or over the outsides of horizontal tubes, and heated gas is moved in counter-current direction to concentrate solutions; such systems are highly engineered at large scales for precise and efficient flow.

Although the spherical falling film EQL and the marble EQL both promote gas exchange on the surface of spherical objects, the function of the two designs is substantively different. The marble EQL uses dozens of small glass marbles to fill a vertical, cylindrical chamber. Marbles maximize the surface area over which water can flow and on which gas exchange takes place. However, the flow of water over and between the marble surfaces is qualitatively different from the flow of water over the single large spherical EQL member. Water introduced above the marbles trickles through the matrix of spherical surfaces and interacts with air moving upward in the opposite direction. Water flow through the marble matrix is chaotic and the water layers generated are far less uniform than a falling film over a single large spherical EQL member. Moreover, the porosity of the marble matrix, generated by the voids among the marbles, is primarily dependent on the diameter of the marbles, but changes as suspended solids and debris are trapped in the matrix, which then change the characteristics and rate of flow. The spherical falling film EQL generates a water layer that is more uniform and consistent through time. The falling film EQL was purposely designed without complex interstices and small diameter passageways to permit sediments and debris to flow freely over the sphere and drain by gravity out a large port.

The extensive body of research and application of falling film technologies provided a theoretical basis for us to explore their use as gas exchange surfaces for gas-liquid equilibration. When water is delivered over the surface of a spherical or substantially spherical surface, it produces a stable falling film for gas exchange. In our design, water flows over the sphere, which is housed in a chamber that provides an airtight outlet for water to drain at or near the bottom. A closed loop air circuit moves air counter-current to the water flow, with an air outlet at the top of the EQL chamber. Humid air is dehumidified before passing across an IRGA, and is pumped back into the bottom of the EQL chamber. An advantage of the closed loop versus open loop configuration is that the same carrier gas is circulated continuously and can track changing EQL conditions rapidly. In contrast, the open loop multi-chamber thin film EQL must fully equilibrate atmospheric air on a single pass. The simplified spherical design lacks the tortuous water routes and small orifices that are integral to other EQL’s design and function; and accordingly, clogging, freezing, biofouling, and flooding are minimized. Controlled stable flow of liquid over the sphere also allows the EQL (and attack angle of water) to be tilted substantially off the vertical axis and still maintain maximal wetted surface area. This is a distinct benefit if the EQL is used in the field or located on a floating platform such as a ship, skiff, buoy, or small autonomous craft. Here, we introduce the design of a simple spherical falling film EQL and discuss its performance based on laboratory and field tests that characterize its sizing, response time, accuracy, and robustness to challenging environmental conditions.

## Materials and methods

We conducted a series of laboratory tests to determine the performance and behavioral characteristics of a spherical falling film gas-liquid EQL. In particular, we investigated if EQLs of various dimensions produce the same equilibration results under identical conditions, as evidence that a true equilibrium was being achieved. The accuracy and precision of the spherical falling film EQL was then tested in a closed tank system containing challenge water that was saturated with certified CO_2_/air gas mixtures standards (S2 Fig). Using the same closed tank system, we also quantified the response time of the spherical falling film EQL by determining the time constant (τ = time for C_t_/C_o_ = 1/e for high to low equilibration and τ = time for C_t_/C_o_ = 1 –(1/e) for low to high equilibration, where C_0_ and C_t_ = concentrations at times 0 and t) using step tests where the EQL was switched between water sources of contrasting pCO_2_. Finally, the spherical falling film EQL was field tested in side by side tests with an open loop multi-chamber thin film EQL to investigate differential performance under very high and very low pCO_2_ conditions in the natural environment.

### Falling film hydrodynamics

Using a 10-in diameter sphere with constant water flow rate (72.55 gal/hr = 4.58 L/min or 76.3 cm^3^/s), we used slow motion video and a timer to measure the average velocity of the falling film. Paper hole punch chads (6 mm diam) were introduced near the top of the sphere, and the elapsed time to travel from top to bottom was recorded and measured. Empirically measured water velocity enabled us to estimate the average residence time of a parcel of water on the spherical surface, the turnover rate, and the average film thickness.

#### Modeling the falling film

To more fully understand the nature of a falling film of water over a spherical surface, (i.e., Reynolds number, film thickness, fluid velocity) we chose to apply a theoretical model for comparison to our observations. We were unable to find a model to expressly characterize the fluid dynamics of a thin falling film over a sphere (as opposed to drag produced by fluid flow past a sphere). However, because a sphere of diameter *d* has the identical surface area as a horizontal tube with diameter = *d* and length = *d*, minus the open ends, (i.e., 4πr^2^ = surface area of a sphere, 2rπ *2r = 4πr^2^ = surface area of a horizontal tube), and because both have the same radius of curvature, we chose to model falling film characteristics of water over a horizontal tube to approximate flow over a sphere of similar diameter. Takagi and Huppert [21] explored the instability of the leading front of falling films, when a discrete volume of fluid was introduced to create a gravity-driven film over horizontal cylinders and spheres. Because their focus was on the characteristics of fingerling rivulet generation during initial wetting of the surface, full wetting of the cylinder and sphere was not investigated. Nevertheless, Takagi and Huppert [21] concluded that although flow over round cylinders is two-dimensional and over spheres axisymmetric, the flows are quite similar, lending further support to our modeling approach. We used the classic equations developed by Nusselt [22–23] and which have been applied more recently by other investigators of horizontal tube falling films [19,24–25].

To calculate the Reynolds film number and estimate the thickness of the falling film, we used Nusselt’s equations (Eq 1 and Eq 2). Eq 3 [26] was used to estimate the mean velocity of the falling film (*v'*).

$$Re=\frac{4\Gamma }{\mu }$$ (1)

$$\delta ={\left[\frac{3\mu \cdot \Gamma }{\rho_{L}(\rho_{L}-\rho_{G})g\cdot sin\beta }\right]}^{1/3}$$ (2)

$$\Gamma =\rho_{L}\cdot v\prime \cdot \delta (\beta )$$ (3)

Where,

Re, film Reynolds film number, [–]

Γ, film mass flow rate on one side per unit length of a cylinder, (0.1502), [kg/m·s]

μ, Dynamic viscosity, (0.001), [kg/m·s]

*β*, angular position on a horizontal tube measured from the top, [°]

*g*, acceleration due to gravity (9.81), [m/s^2^]

ρ_L_, Density of water, (998.02), [kg/m^3^]

ρ_G_, Density of air, (1.225), [kg·m^3^]

*δ*, film thickness as a function of *β*, [m]

*v'*, mean water velocity, [m/s]

### Comparative performance using spherical EQLs of different sizes

Spherical falling film EQLs of a variety of sizes: 10.0 in (25.4 cm), 8.0 in (20.3 cm), 6.0 in (15.2 cm), and 3.7 in (9.4 cm) were compared to determine whether the degree of equilibration was a function of wetted surface area and/or EQL chamber volume. Tests were carried out as pair-wise contrasts. The first comparison was between an EQL consisting of two vertically stacked 10-in diameter spheres inside a single large chamber and a single 10-in sphere inside a chamber of one half the volume. A second comparison was between EQLs with 10-in and 8-in spheres in separate chambers. Finally, pairwise comparisons were made between EQLs with 8-in, 6-in and 3.7-in spheres. Table 1 presents details on dimensions of all the EQL members and chambers used in these experiments.

**Table 1:** Dimensions of four spherical equilibrators tested.

Diameter (cm)	Surface Area (cm^2^)	Headspace (cm^3^)	EQL Air turnover (min) @ 1 LPM flow
3.7 in (9.4)	278	476	0.476
6.0 in (15.2)	726	1,671	1.671
8.0 in (20.3)	1,295	3,096	3.096
10.0 in (25.4)	2,027	4,443	4.443
2 x 10.0 in (25.4)	4,054	8,886	8.886

We used a 400 L water tank with semi-enclosed top outfitted with a submersible water pump (S2 Fig). Water was pumped continuously from the bottom of the tank, through a manifold with flow controllers, and into the water intake ports of paired EQLs. Both EQLs drained back into the tank. Headspace air was continuously circulated (1 L/min flow) with a small diaphragm pump from the headspace of the EQL through two IRGAs in series (a Senseair K30 with nominal accuracy ± 30 ppmv ± 3% of reading, range = 0–30,000 ppmv and a Licor LI-7000 with nominal 1% accuracy, range = 0–3000) [27–28] and back into the base of the EQL. Note: we report all experimental measurements of CO_2_ concentration as the raw dry air mixing ratio = xCO_2_ throughout this study. The 400 L tank was also outfitted with a large air stone to allow the water to be enriched with compressed CO_2_ or scrubbed gradually by bubbling with CO_2_-stripped air. This allowed for testing at a variety of different CO_2_ concentrations, from sub-atmospheric up to ~6500 ppmv. Water temperature was monitored continually at 1-min intervals, as was barometric pressure and relative humidity inside each IRGA chamber. The air lines were valved so that head space sampling could be switched rapidly between EQLs. Because water was pumped simultaneously and continuously across both EQLs, gas invasion and evasion between EQL chamber and room atmosphere was precluded and startup lag (dead time) was minimized, allowing for precise comparisons. This method was modified for the 10-in to double 10-in EQL comparison and when the 3.7-in EQL was swapped for a 6-in EQL when comparing with 8-in EQL, where a single water hose and set of air lines were swapped from one to the other.

Measurements for the higher precision IRGA (the Licor LI-7000) were reported for the first two comparisons, because concentrations were largely within its working range. During the third experiment (8-in vs 6-in vs 3.7-in EQLs), upper CO_2_ concentrations reached above 6000 ppmv and we report measurements from the Sensair K30 IRGA; however, these values were compared with those of the corresponding LI-7000 to examine the differential performance of these IRGAs. Martin et al. [29] evaluated the Sensair K30 and found that the manufacturer’s stated accuracy can be improved to < 5 ppmv, or approximately 1% of the observed value, when corrected for sensitivity to temperature, relative humidity, and pressure. It should be noted that although we calibrated our K30 sensor, and measured temperature, relative humidity, and pressure at the sensor, we applied no correction for the data reported in this study.

### Spherical falling film–Accuracy measurements through equilibration with standard gas mixtures

In these tests, a 5-gal (18.9 L) polypropylene bucket with a screw-on airtight lid was plumbed with airtight fittings to the EQL chamber, allowing water to be circulated between the two via pumping and draining without contamination from room air (S2 Fig). The bucket was filled with tap water and enriched for one to two hours with one of two certified CO_2_ gas mixtures, prior to equilibration testing. The standards tested were 1047 ppmv or 7579 ppmv CO_2_ gas mixture (±1% uncertainty, Roberts Oxygen Specialty Gas Lab, Gaithersburg, MD). The EQL used for these tests was a 3.7-in (9.4 cm) diameter sphere. Water was pumped at a rate of 99.85 gal/h (6.31 L/min) with a small 12v water pump into the top of the EQL chamber, over the sphere, and drained back into the bucket in a closed loop. At this pump rate, the entire 5-gal volume of water was turned over approximately once per 3 mins. Air was circulated in a closed loop between the EQL and the IRGA by a small 12v diaphragm pump at a rate of 1000 mL/min, producing a water:air pumping ratio = 6.31:1. The volume of EQL headspace (V_HS_ = 476 cm^3^) was determined by measuring EQL container volume (V_EQL_ = 1420 cm^3^) and subtracting the volumes of EQL sphere (V_SP_ = 435 cm^3^) and steady state pooled water in the running EQL (V_H20_ = 509 cm^3^). Humidity was controlled by a small water trap and Nafion^™^ tubing. Air and water circulated continuously and xCO_2_ was measured and recorded once per minute, as were barometric pressure, relative humidity and temperature inside the IRGA chamber. A temperature probe measured and logged the water temperature in the bucket. The IRGA used in these tests was a Sensaire K30 sensor with a working range of 0 to 30,000 ppmv. Time series equilibration measurements were plotted in relation to target standard gas mixtures and the uncertainties associated with the standard gas and published sensor accuracy. Prior to testing, the K30 IRGA was calibrated using a zero gas as per manufacturer’s protocol and checked with span gases (1047 ppmv and 7579 ppmv, the same as used for the EQL accuracy test).

### Response time–Time constant determination

The EQL was plumbed in the same closed loop configuration (S2 Fig) as described above for the EQL accuracy tests and used the same K30 IRGA. For these experiments, the air flow was set at 250 mL/min and water flow set at 6.31 L/min. Pairs of 5-gal buckets of tap water were prepared with contrasting xCO_2_ challenge conditions (from 80 to 130 ppmv in “low” buckets to 1000 to ~50,000 ppmv in “high” buckets) to measure equilibration response times across a wide variety of concentration gradients. The EQL was swiftly shifted between two volumes of water to measure rates of equilibration, upward and downward. We experienced no measurable perturbation from room air contamination as described by Gülzow et al. [30] and Webb et al. [13] in their open tank tests.

To formally assess the response time of the falling film EQL, we calculated the time constant (τ) for high to low concentration responses over several periods of 1/e decay in concentration (nτ = time at which C_t_/C_0_ = 1/e^n^; e.g., 3τ = time when C_t_/C_0_ = 1/e^3^), and for low to high responses, (nτ = time when C_t_/C_0_ = 1-1/e^n^). Here, n represents the number of sequential periods of 1/e decline in concentration driving force for transfer and τ is sometimes referred to as the e-folding time in a process for which there are n “e-folds.” The system was turned off for 1–2 mins between trials in which the EQL was swiftly moved between water volumes. In this configuration, the dead time of the EQL/IRGA, or time for the EQL/IRGA to initially detect measurable change in xCO_2_, was determined to be < 1 min. Raw xCO_2_ values were scaled by subtracting the minimum measured concentration from all measurements within a trial prior to calculating time constants, τ (mins). For high to low (descending) equilibration trials, τ values were calculated at concentration ratios of 1/e^1^, 1/e^2^, 1/e^3^, 1/e^5^, where exponents determine the degree of decay from 36.8% to 0.7% of maximum starting concentration. For low to high (rising) equilibration trials, τ values were calculated as 1-1/e^1^, 1-1/e^2^, 1-1/e^3^, 1-1/e^5^ and represent rates of increase of 63.2% to 99.3% of the maximum equilibrated value.

### Falling film sphere vs multi-chamber thin film EQL–Field comparisons

Side by side comparisons of a 10-in spherical, closed loop EQL and Poisson-type multi-chamber thin film, open loop EQL (S3 Fig) were made in two field locations on the Rhode River, a subestuary of the Chesapeake Bay (Edgewater, MD USA). The river water at the first location, the Smithsonian Environmental Research Center’s (SERC) pier, is characterized by strong diel cycling due to differential photosynthesis and respiration in warm months. In contrast, the reduced respiration in cold months produces periods of steadier sub-atmospheric pCO_2_ (see Results). The second location, also adjacent to SERC property, was the tidal creek that feeds and drains the Kirkpatrick Marsh, a small pocket marsh on the Rhode River, which is dominated by the tidal cycle and characterized by very high pCO_2_ at low tide when CO_2_ from root respiration and other wetland processes [31] is flushed into the river. The two Senseair K30 IRGAs were calibrated and the EQLs arranged side by side at equal water and air flow rates to compare performance. Data were measured once per min over two days.

### Ethics statement

All laboratory experiments and field observations were made on the property and within the facilities owned by the Smithsonian Environmental Research Center (SERC), Edgewater, MD USA. All research was carried out in accordance and in compliance with the legal and ethical guidelines required by the Smithsonian Institution. No animals, endangered species, protected species, or otherwise were included or affected by this research.

## Results

### Falling film hydrodynamics

#### Empirical observations

At all flow rates and sphere diameters tested, the entire volume of water introduced at the top of a sphere clings to the sphere and falls downward, wetting the entire surface of the sphere before releasing at the bottom, or where the film contacts pooled water at the floor of the EQL chamber. Pumped water is thoroughly converted into a falling film where gas exchange can take place. With adequate flow, the falling film remains fully intact even when the water’s angle of attack is tilted up to 45 degrees off the vertical, indicating that the falling film over a sphere is quite stable and resistant to physical perturbation, enabling the EQL to be deployed easily and reliably under many field conditions. Slow motion video reveals a complex wavy structure of the falling film.

To estimate the velocity of the thin film of water traveling over the spherical surface of the 10-in diameter EQL, the elapsed time for 6-mm diameter paper chads (dropped onto the surface at the top of the sphere) to travel over a fixed distance was measured with slow motion video and timer under a constant flow rate (72.55 gal/h = 76.3 cm^3^/s). Ten trials determined that the average elapsed time to travel across an 11.5 in (29.21 cm) section of the spherical surface = 0.291s ± 0.09 s (mean ± 1 SD), equaling a mean velocity of 100.3 cm/s (29.21 cm/0.291 s). At this velocity, a parcel of water appears to traverse from the top to the bottom of the sphere, a distance of 39.9 cm, in 0.4 s, the equivalent of 2.51 surface turnovers/s.

From the turnover rate (2.51/s) and surface area of the sphere (2027 cm^2^), the total wetted surface area produced per second can be calculated (= 5088 cm^2^/s). The average instantaneous water thickness over the surface of the sphere is coarsely estimated by dividing the water flow rate by the total surface area wetted per second (76.3 cm^3^/s)/(5088 cm^2^/s) = 0.015 cm, or 0.15 mm).

#### Nusselt model

Using the delivery flow rate of water to the sphere = 76.3 cm^3^/s, we calculated the film mass flow rate over one-half of a 10-in sphere per unit length (i.e., multiplying the mass flow rate over a single sphere by 1 m/0.254 m = 3.93) as 0.1502 kg/s·m. The falling film thickness at 90° from the top of the sphere (at the equator) = 3.6 x 10^−4^ m or 0.36 mm (Eq 1). The Reynolds film number, Re, was calculated as 601 (Eq 2). These results comport well with other studies that have examined falling films of water over horizontal tubes at similar mass flow rates [32–33]. In their review of falling film evaporation on horizontal tubes, Ribatski and Jacobi [20, and research reviewed therein] indicate that Re > 6000 corresponds to fully turbulent flow. Brumfield and Theofanous [34] suggest that flow of a falling film over a horizontal tube has two regions, the base film that is in direct contact with the tube surface and waves above, and that the wave structure may partially control the underlying base film flow. Thus, at low Re values, both regions exhibit laminar flow. At intermediate Re, the base is laminar and the waves are turbulent, and at high Re, both regions are turbulent. For flow over a horizontal tube, Carey [35] recommended a transitional Re = 1500. Under these criteria, a Reynolds film number of 601 would be categorized as fully laminar flow.

For the purposes of modeling the falling film velocity, we used the calculated value of 0.36 mm as an average film thickness (i.e., the Nusselt thickness); rearranging Eq 3, water surface velocity over the sphere = 0.42 m/s. Our empirically measured water velocity was 1.03 m/s, about 2.45 times the theoretical value. One possible explanation for this divergence is that the paper chads we used to determine velocity travelled on the outer surface of the falling film (the wavy region), and that velocity nearer the sphere’s surface was slowed due to the water’s interaction with the sphere’s surface. Parabolic velocity profiles, where flow at the liquid wall interface is slowed and increases with distance from the inner wall of a pipe are well known and these velocity differences are accentuated under laminar flow [36]. Mudawar and Houpt [33] measured falling film velocity and dynamics using laser doppler velocimetry, and confirmed that falling films tend not to accelerate because the viscous shear balances the gravitation body forces in fully laminar falling films. Kumar [25] showed that film thickness increases with Re and that Re and film thickness in the vicinity those modeled here (601 and 0.36 mm) are reasonable approximations.

Setting the velocity of water over the sphere = 0.42 m/s, water will traverse from the top to the bottom of the 10-in sphere in 0.95 s, rewetting the entire surface 1.05 times/s. Thus, the total wetted surface generated per second = 1.05/s x 2027 cm^2^ = 2128.35 cm^2^/s. Using the simple conservation of mass principle, the average falling film thickness can be estimated as (76.3 cm^3^/s)/(2128.35 cm^2^/s) = 0.036 cm, or 0.36 mm. This value agrees with that derived from Eq 1 at an angular position = 90º, suggesting that our estimate of the mass flow rate (0.1502 kg/m·s) is reasonable and that the use of the horizontal tube falling film model applies well to spherical falling films.

### Comparative performance of different sized spherical EQLs

**Fig 2:**
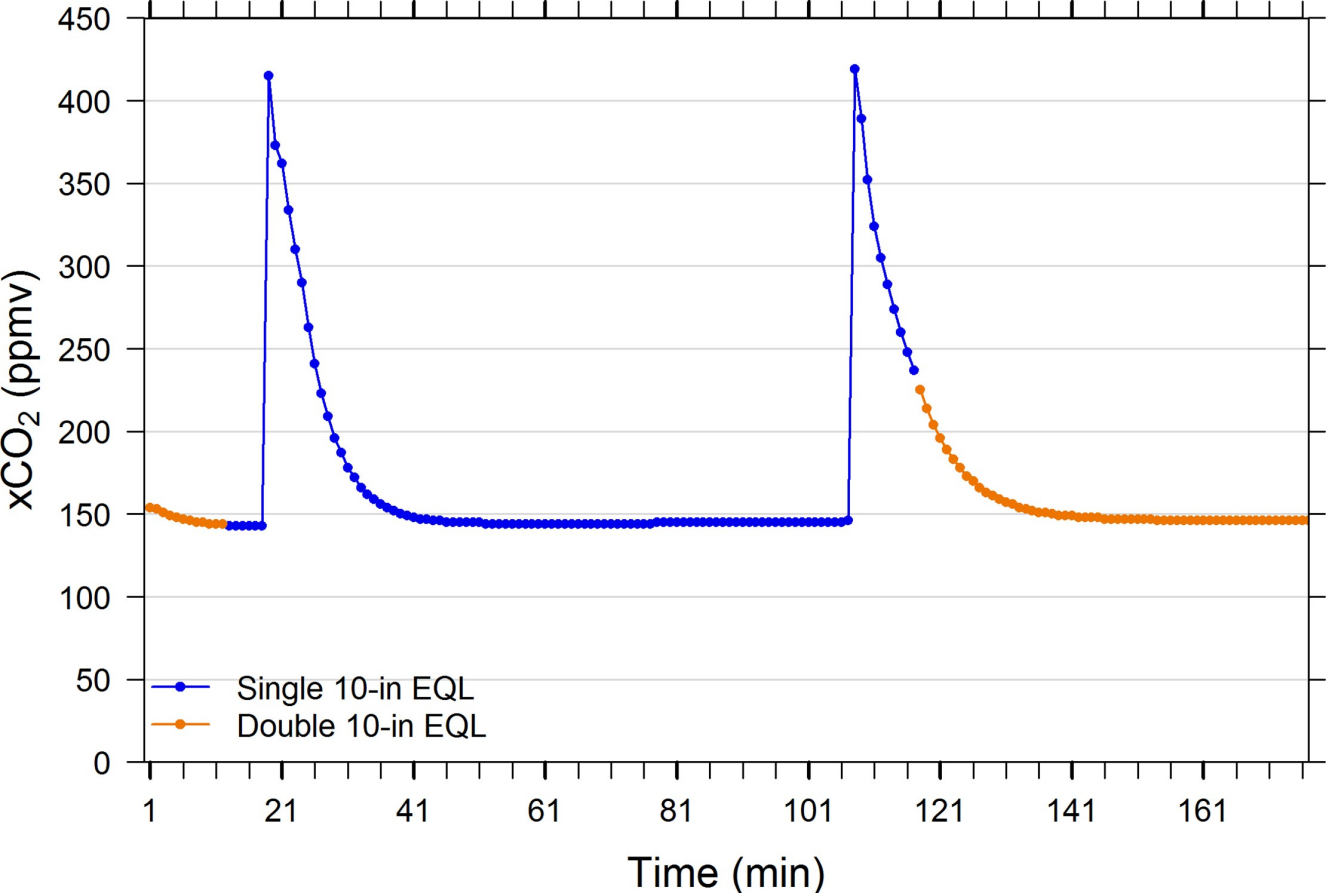
Performance comparison of a single 10-in diameter spherical equilibrator with an equilibrator consisting of two stacked 10-in diameter spheres in a large chamber. See Table 2 for EQL specifications. Note: see Supporting Information (S1–S11 Tables) for all data relevant to Figs 2–12.

**Fig 3:**
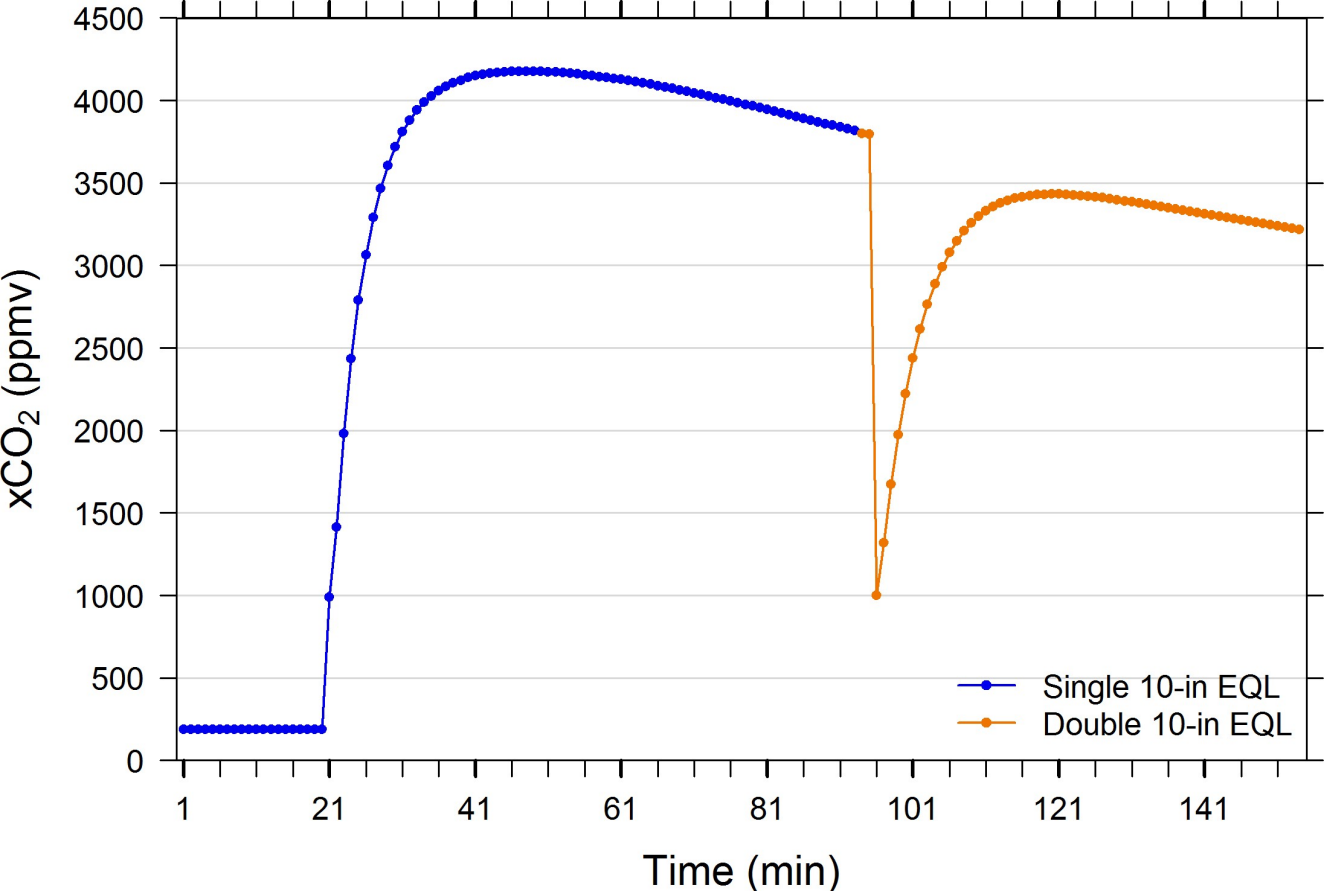
Performance comparison of a single 10-in diameter spherical equilibrator with a two stacked 10-in diameter spheres in a large chamber. Water hose and sample air lines to air pump and IRGA circuit were rapidly switched manually between EQLs for comparison. Water tank was enriched with compressed CO_2_ at min 20. The single 10-in EQL was swapped for the double 10-in EQL (containing ambient room air) at min 94 –note 2-min dead time prior to sensor response. See Table 2 for EQL specifications.

Pairwise comparisons of EQLs performance at equal water and air flow rates indicate that similar levels of equilibration were achieved regardless of EQL sphere diameter/surface area and chamber volume. Thus, within the range of EQL sizes investigated (3.7-in to two stacked 10-in diameter spheres, Table 1), all reached similar degrees of equilibration across a broad range of xCO_2_ conditions. Figs 2 and 3 reveal that a single 10-in diameter EQL equilibrates to the same degree as two stacked 10-in spheres inside a single chamber at sub-atmospheric and between 3000 and 4000 ppmv. The size of the EQL chamber (i.e., headspace volume) will affect the rate of response because the gas turnover time is greater for larger chambers (Table 1). Indeed, Fig 3 suggests that the time to reach equilibration is approximately twice as long in the double 10-in EQL (e.g., the time to equilibrate from 1000 to 3000 ppmv is 5 mins for the single 10-in EQL and 10 mins for the double 10-in EQL), demonstrating the importance of headspace volume and air flow rate.

**Fig 4:**
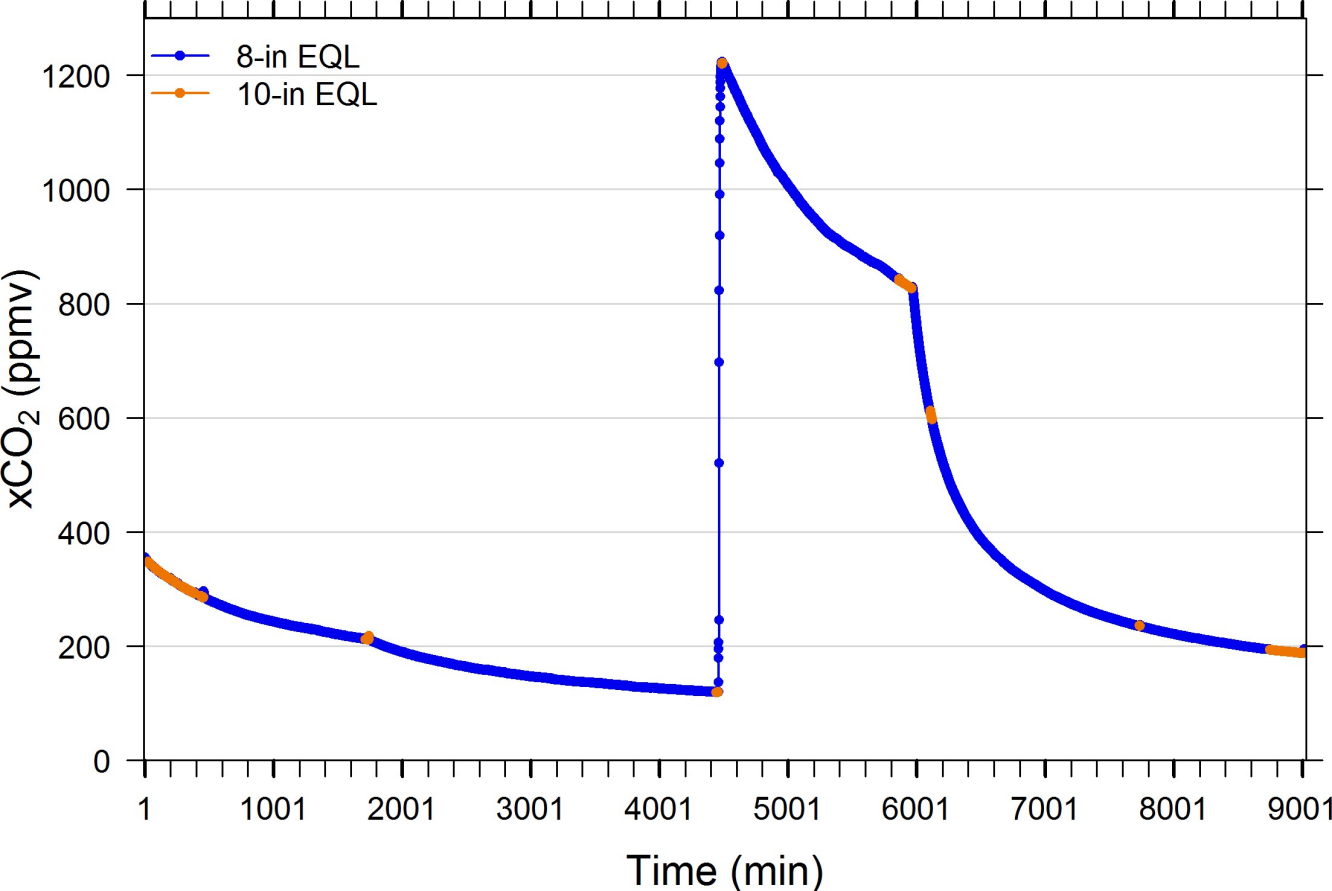
Performance comparison of 10-in and 8-in diameter spherical equilibrators. Common water was pumped continuously to both EQLs throughout the 6-day period and valves connecting sample lines to air pump and IRGA circuit were switched periodically for instantaneous comparisons. Changes in slope are due to increased bubbling of test tank with CO_2_-stripped air. Tank was enriched with compressed CO_2_ at minute 4457. See Table 2 for EQL specifications.

**Fig 5:**
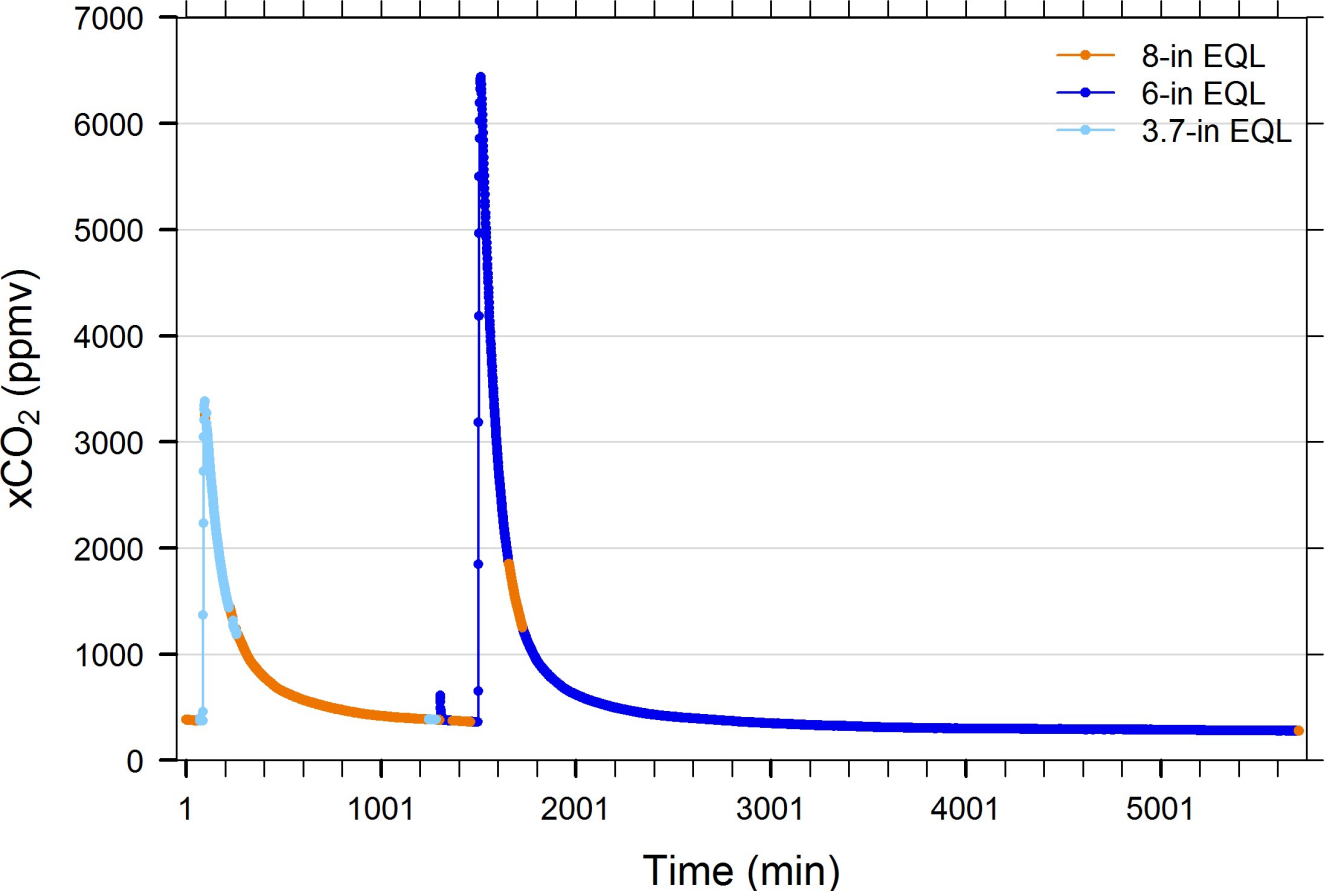
Performance comparison of 8-in, 3.7-in, and 6-in diameter spherical equilibrators. Common water was pumped continuously to selected pairs of EQLs throughout the 4-day period and valves connecting sample lines to air pump and IRGA circuit were switched periodically for instantaneous comparisons. Changes in slope are due to increased bubbling of test tank with CO_2_-stripped air. Tank was enriched with compressed CO_2_ at minute 1500. Note: 3.7-in EQL was replaced with 6-in EQL at minute 1304, as indicated by small spike caused by brief non-equilibrium starting condition of 6-in EQL. See Table 2 for EQL specifications.

Fig 4 indicates that when the air lines connecting the IRGA to the EQL inlet and outlet were arbitrarily swapped from a 10-in to an 8-in diameter EQL and vice versa, that both yielded the same xCO_2_ values. Likewise, when a 3.7-in, 6-in, and 8-in diameter EQLs were compared serially, all yielded the same xCO_2_ result (Fig 5).

### Falling film sphere–Accuracy equilibrating standard gas mixtures

**Fig 6:**
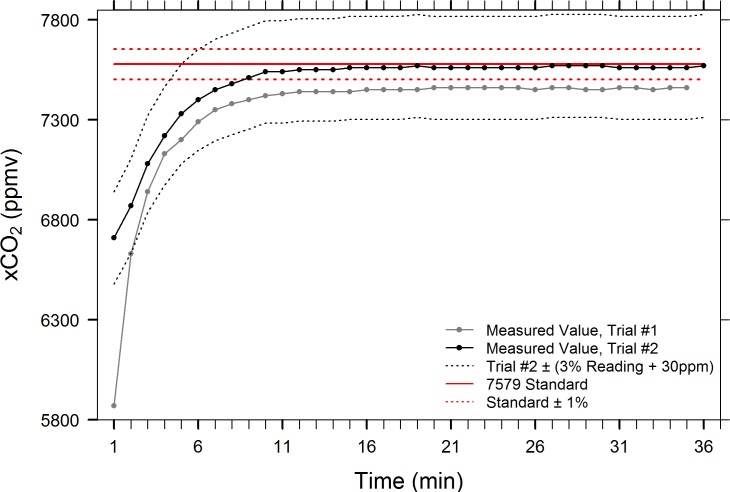
Accuracy test of 3.7-in EQL using 7579 ppmv ± 1% (nominal CO_2_ concentration). Test #1 failed to reach the 7579 ppmv target, reaching an asymptote Accuracy test of EQL using 7579 ppmv ± 1% (nominal CO_2_ concentration). Once equilibration initially reached within 1% of standard gas concentration, the average xCO_2_ = 7578 ± 12.2 (mean ± 1 SD, n = 29).

**Fig 7:**
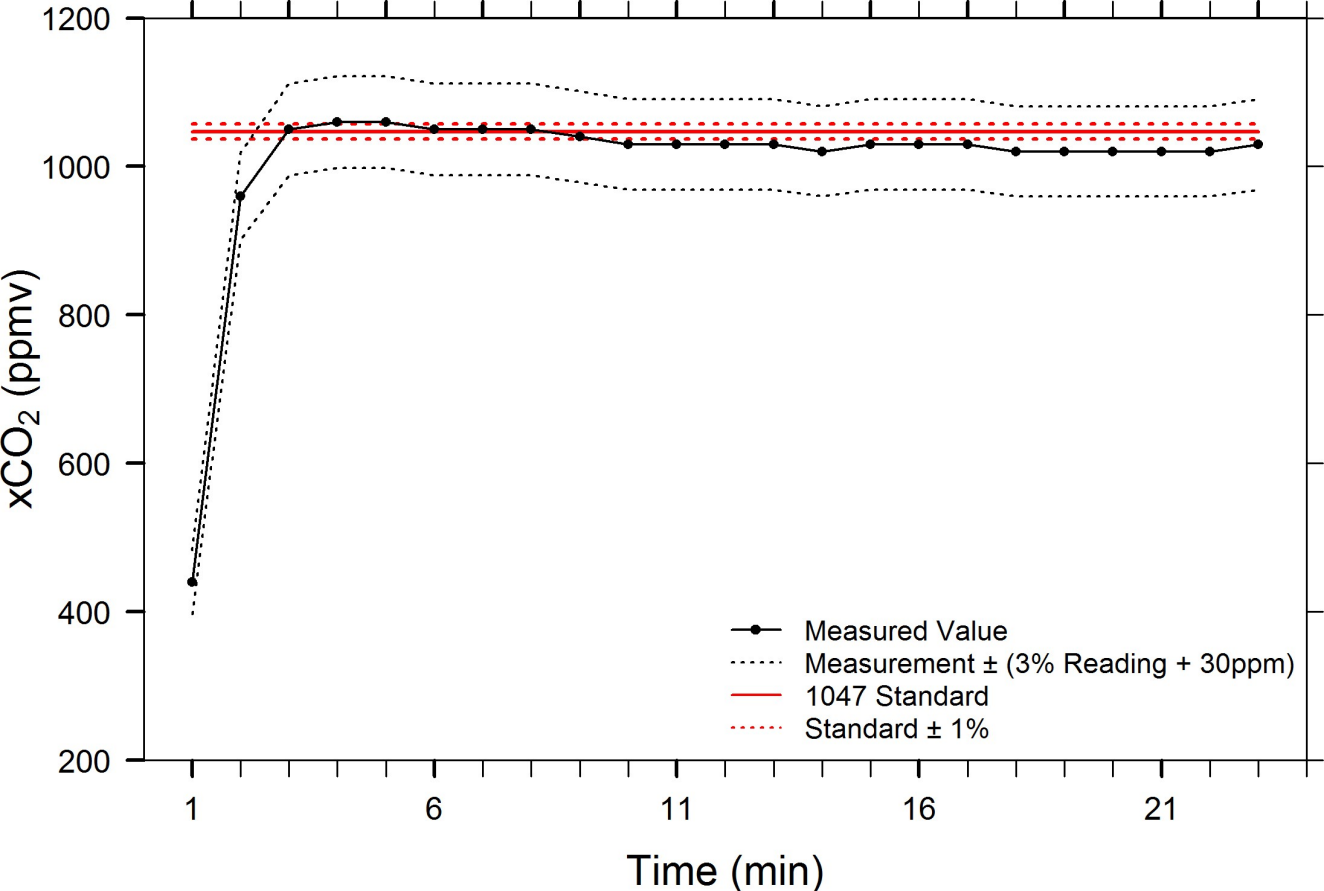
Accuracy test of EQL using 1047 ppmv ± 1% standard (nominal CO_2_ concentration). Once equilibration initially reached within 1% of standard, the average xCO_2_ = 1034 ± 13.6 (mean ± 1 SD, n = 21).

We tested the 3.7-in diameter falling film EQL against water that was saturated with one of two certified standard CO_2_/air mixtures, 1047 ppmv and 7579 ppmv ± 1%. Two trials were conducted using the 7579 ppmv standard. During trial #1, the starting xCO_2_ value in the IRGA chamber was measured at 480 ppmv and the equilibration failed to reach within the ± 1% uncertainty of the standard gas, instead attaining a stable asymptote of 7453 ± 7.6 ppmv (mean ± 1 SD, min/max = 7440/7460, CV = 0.102%, n = 24, Fig 6). To rule out the possibility that the challenge water was not fully saturated, the test bucket was enriched again with the standard gas for one additional hour prior to a second trial. During trial #2, equilibration within 1% of the standard was achieved by min 9 and remained at a stable asymptote of 7560 ± 8.2 ppmv (mean ± 1 SD, min/max = 7540/7570, CV = 0.106%, n = 28, Fig 6) for the remainder of the trial. A separate trial using a 1047 ppmv ± 1% standard enriched challenge water revealed similar results, but with a somewhat less stable asymptote of 1034 ± 13.6 ppmv (mean ± 1 SD, min/max = 1020/1060, CV = 1.32%, n = 21, Fig 7). The xCO_2_ value in the IRGA chamber and EQL air circuit was sub-atmospheric (240 ppmv) at the start of the trial, and it is unclear whether the results of this test may have been partially influenced by dilution, given the relatively small volume of water being tested. Despite having a coarser manufacture’s accuracy rating than the LI-7000 (3% of instrument reading ± 30 ppmv vs. 1% of reading), the SenseAir K30 sensor accurately measured the narrow target range (1047 ppmv ± 10 ppmv) of this standard. These results indicate that the 3.7-in EQL, and by extension, larger spherical falling film EQLs, can fully and accurately equilibrate water with the air in the EQL head space.

### Response rate–Time constant determination

**Fig 8:**
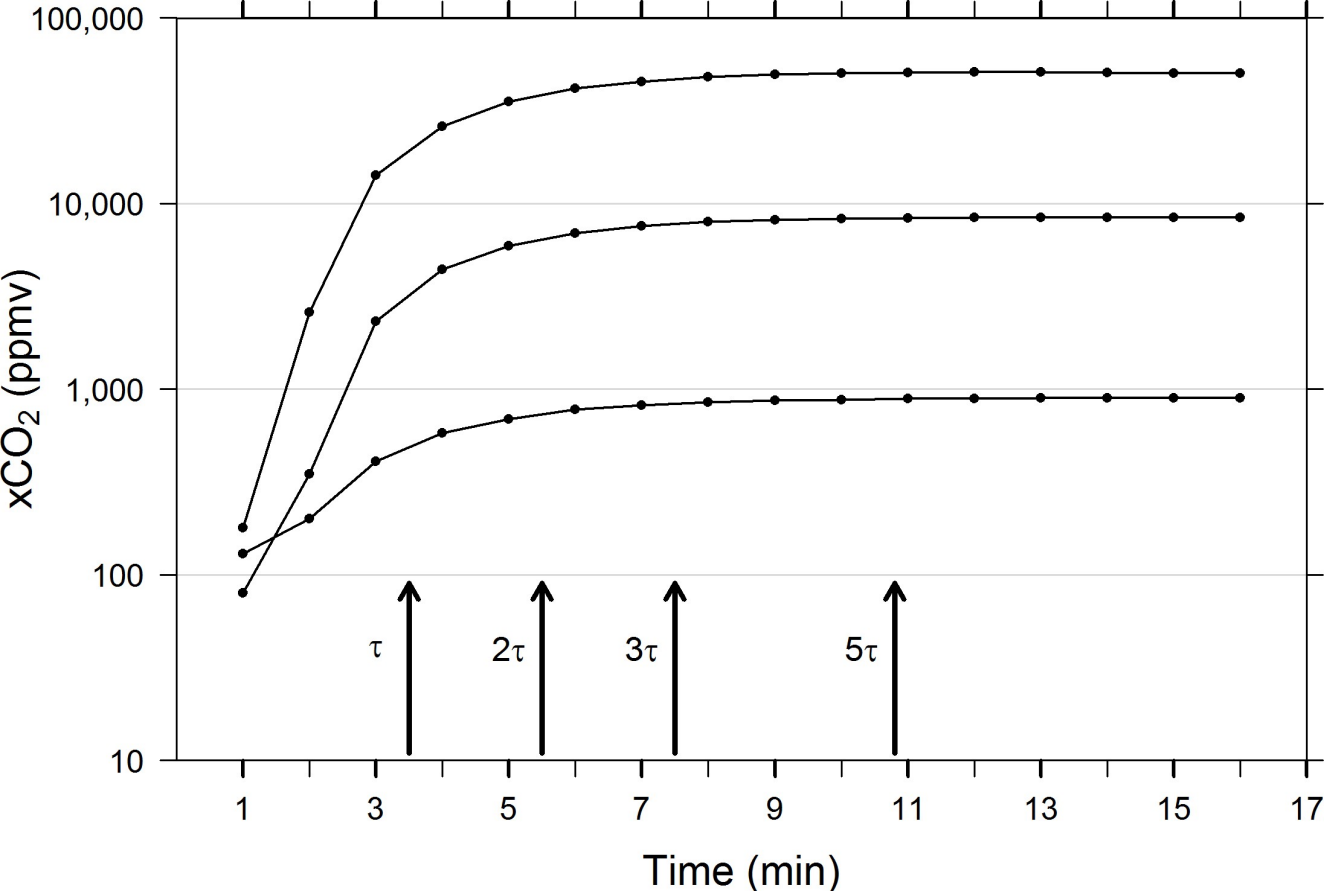
Results from three low to high concentration equilibration trials. Multiples of τ (mins) correspond to 63.2%, 86.5%, 95.0%, 99.3% of maximum values measured. Arrows indicate mean τ values for all three trials (see Table 1 for summary of mean and standard deviation values). Concentrations are plotted on a log scale to better visualize low and high ranges.

**Fig 9:**
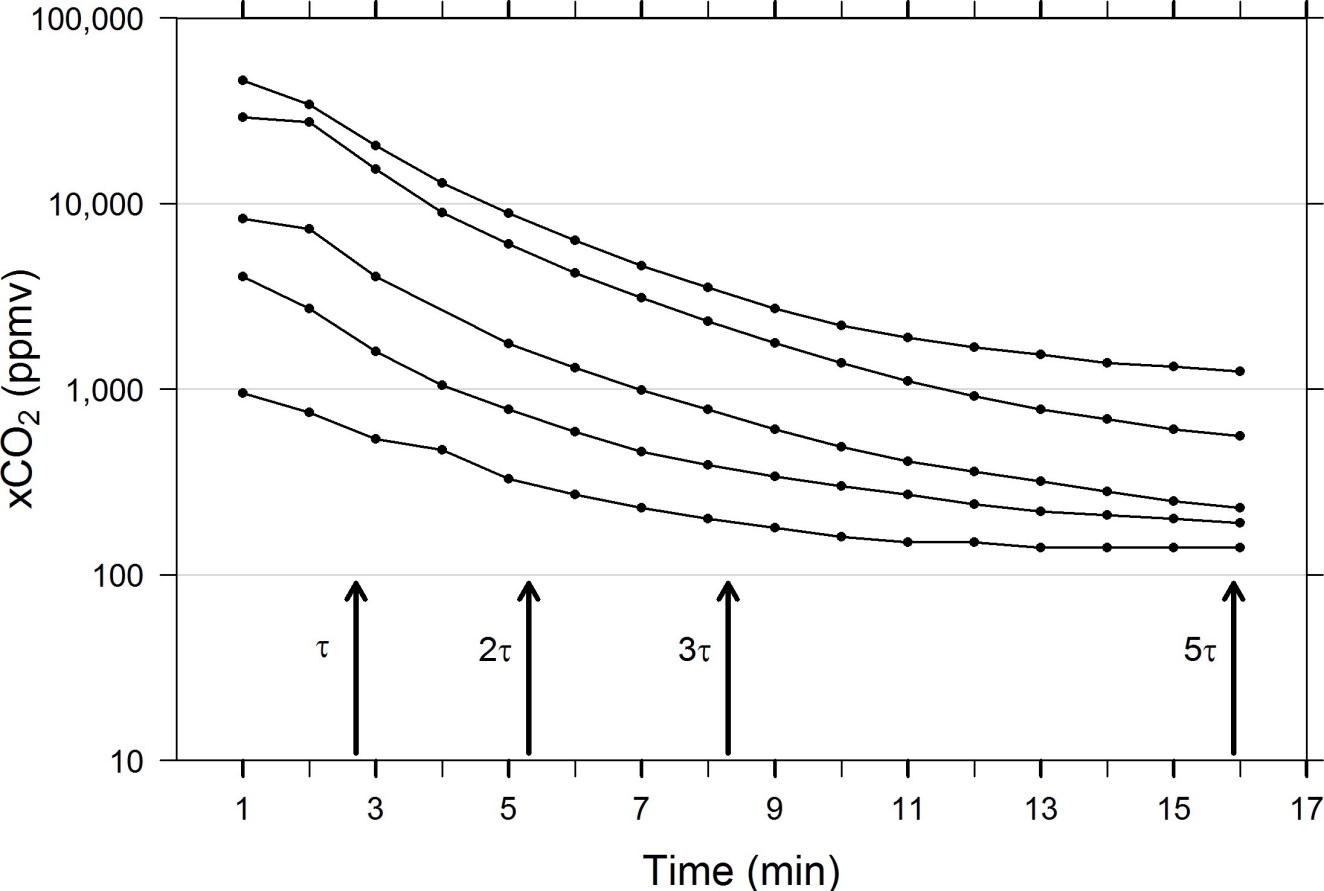
Results from five high to low concentration equilibration trials. Multiples of τ (mins) correspond to 36.8%, 13.5%, 5.0%, 0.7% of maximum values measured. Arrows indicate mean τ values for all five trials (see Table 1 for summary of mean and standard deviation values). Concentrations are plotted on a log scale to better visualize low and high ranges.

Not surprisingly, the absolute rate of equilibration is fastest when concentration gradients are steepest, slowing as gas and water phase concentrations approach one another. This is observed for both rising and descending equilibration functions (Figs 8 and 9). Despite vastly different pairs of starting and ending concentrations, equilibration occurs at fairly similar rates across the first three time constants (τ, 2τ, 3τ, i.e., 63.2%, 86.5%, 95.0% equilibration) in rising and descending equilibrium processes (Figs 8 and 9), but rising equilibration was less variable than descending equilibration for all time constant values measured, as quantified by CV (see Table 2). However, the overall rate of equilibration slows more in shallow xCO_2_ gradients at low concentrations than elevated conditions. In rising equilibration, 5τ (time required to reach 99.3% of maximum value) = 10.8 ± 0.58 min (mean ± 1 SD, n = 3), compared with descending equilibration where the 5τ (time required to reach 0.07% of maximum value) = 15.9 ± 1.14 min (mean ± 1 SD, n = 5, Table 2). Presumably, even when concentrations gradients are proportionally similar, a lower overall concentration of reactant molecules slows the rate of equilibration. When the time constants were calculated from accuracy test trials against known standards, at air flow rates of 1L/mi rather than 250 mL/min, the 7579 ppmv standard trial reached 99.3% equilibration after 9.5 min, slightly faster than mean 5τ at lower air flow. However, the 1047 ppmv trial reached 5τ after just 3.5 mins, substantially faster than the mean 5τ. These results suggest that air flow rates are integral to EQL response time.

**Table 2:** Summary of time constant, τ, for rising and falling equilibration.

Equilibration Direction	Trials (n)	τ (min)	2τ (min)	t_90%_ (2.3τ) (min)	3τ (min)	5τ (min)	
**Rising**	3	3.5 **±** 0.00	5.5 **±** 0.00	6.5 **±** 0.00	7.5 **±** 0.00	10.8 **±** 0.58	**Mean ± SD**
		0.00%	0.00%	0.00%	0.00%	5.37%	**CV**
**Descending**	5	2.7 **±** 0.45	5.3 **±** 1.30	6.3 **±** 1.30	8.3 **±** 1.30	15.9 **±** 1.14	**Mean ± SD**
		16.7%	24.5%	20.7%	15.7%	7.36%	**CV**

Mean time constant, τ ± 1 SD (min) and coefficient of variation (CV%) of rising (τ = 1-1/e) and descending (τ = 1/e) equilibration processes using a 3.7-in (9.0 cm) spherical falling film EQL. CO_2_ concentrations measured varied between a minimum of 80 and maximum of 51,000 ppmv. Water flow rate = 6.31 L/min and air flow = 250 mL/min.

Response times for a variety of air-water EQLs measuring CO_2_ were summarized from the literature by Webb et al. [13] and Yoon et al. [1]. Drawing on the summary of Webb et al. [13], we selected the active equilibration methods that were most comparable to the spherical falling film EQL, and also included the rotating disc of Sabine and Key [16], (Table 3). For marble and showerhead (including Weiss-type) EQLs, response times were reported inconsistently among studies, with investigators using a variety of conventions to report time constants, making direct comparisons somewhat complicated. For example, 6/13 studies reported τ (63%), 4/13 reported 3τ (95%), and 3/13 reported time to reach 90% of maximum value (i.e., approximately 2.3τ). Nevertheless, the τ, 2.3τ, 3τ measurements for the spherical falling film EQL fell somewhere in the mid-range of corresponding time constants reported for each EQL type (Table 3). In our investigation, no efforts were made to optimize the response time of the 3.7-in EQL, so there is likely scope for shortened time constants via water and air flow balancing, as well as minimization of EQL headspace.

**Table 3:** Response times for measuring pCO_2_ in water by active air-water equilibration (after Webb et al. [13]).

	Response Time (min)			Flow		
EQL Type	1 τ (t_63%_)	2.3 τ (t_90%_)	3 τ (t_95%_)	Water (L/min)	Air (mL/min)	Reference
Marble		1.08		3	-	Frankignoulle et al. 2001[9]
Marble		10–12		5.4	1000	Santos et al. 2012 [2]
Marble			2.08			Yoon et al. 2016 [1]
Marble	1.0		2–5.8	1.5–9		Webb et al. 2016 [13]
Showerhead		8–13		2.8	1000	Santos et al. 2012 [2]
Showerhead (rising)	1.2			0.25–2.5	1000–1200	Körtzinger et al. 1996 [37]
Showerhead	0.59					Crawford et al. 2015 [38]
Weiss			2–7	1.5–9		Webb et al. 2016 [13]
Weiss			6	3	100	Friedrichs et al. 2010 [39]
Weiss			12	13–20	6000	Johnson et al. 1999 [15]
Bubble	3.8			0.5	450	Gülzow et al. (2011) [30]
Rotating Disc	1.0					Sabine and Key (1996) [16]
Spherical Falling Film*	3.0	6.4	8.0	6.31	250	Miller et al. (this study)

* Response times from spherical falling film EQL are averaged for both rising and descending equilibration.

### Falling film sphere vs multi-chamber thin film EQL–Field comparisons

**Fig 10:**
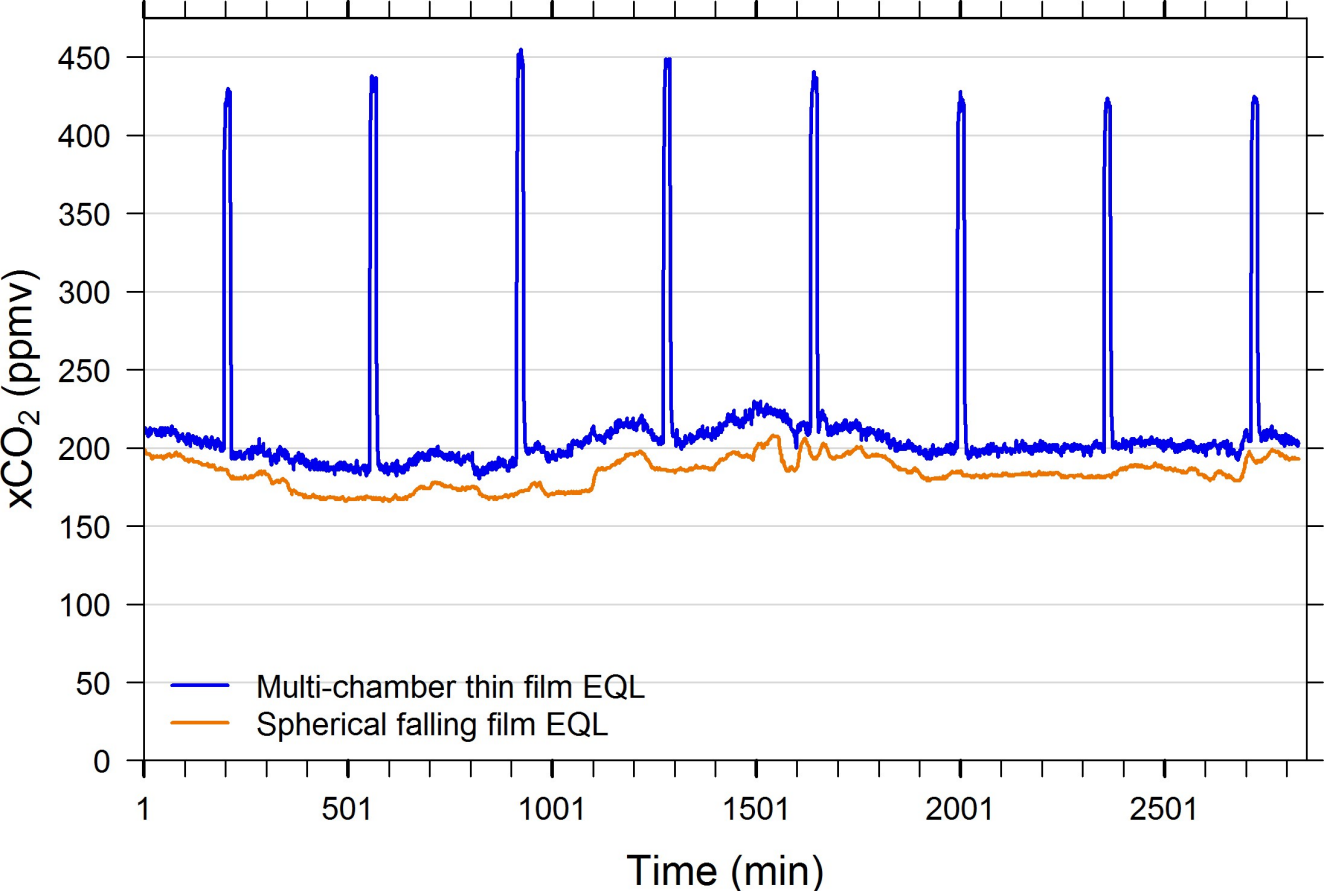
Side-by-side field measurements of multi-chamber thin film open loop and 8-in spherical falling film closed loop EQLs. Measurements were made autonomously at 1-min intervals February 14–16, 2017 in the Rhode River from the SERC pier, Edgewater, MD. The multi-chamber thin film EQL was programmed to sample atmospheric air for 15 mins at 6-hr intervals. The spherical falling film EQL consistently measured lower values than the multi-chamber thin film EQL throughout the time period (mean difference ± SD = -20.3 ± 20.2 ppmv, n = 2707, when readings are excluded during atmospheric readings).

**Fig 11:**
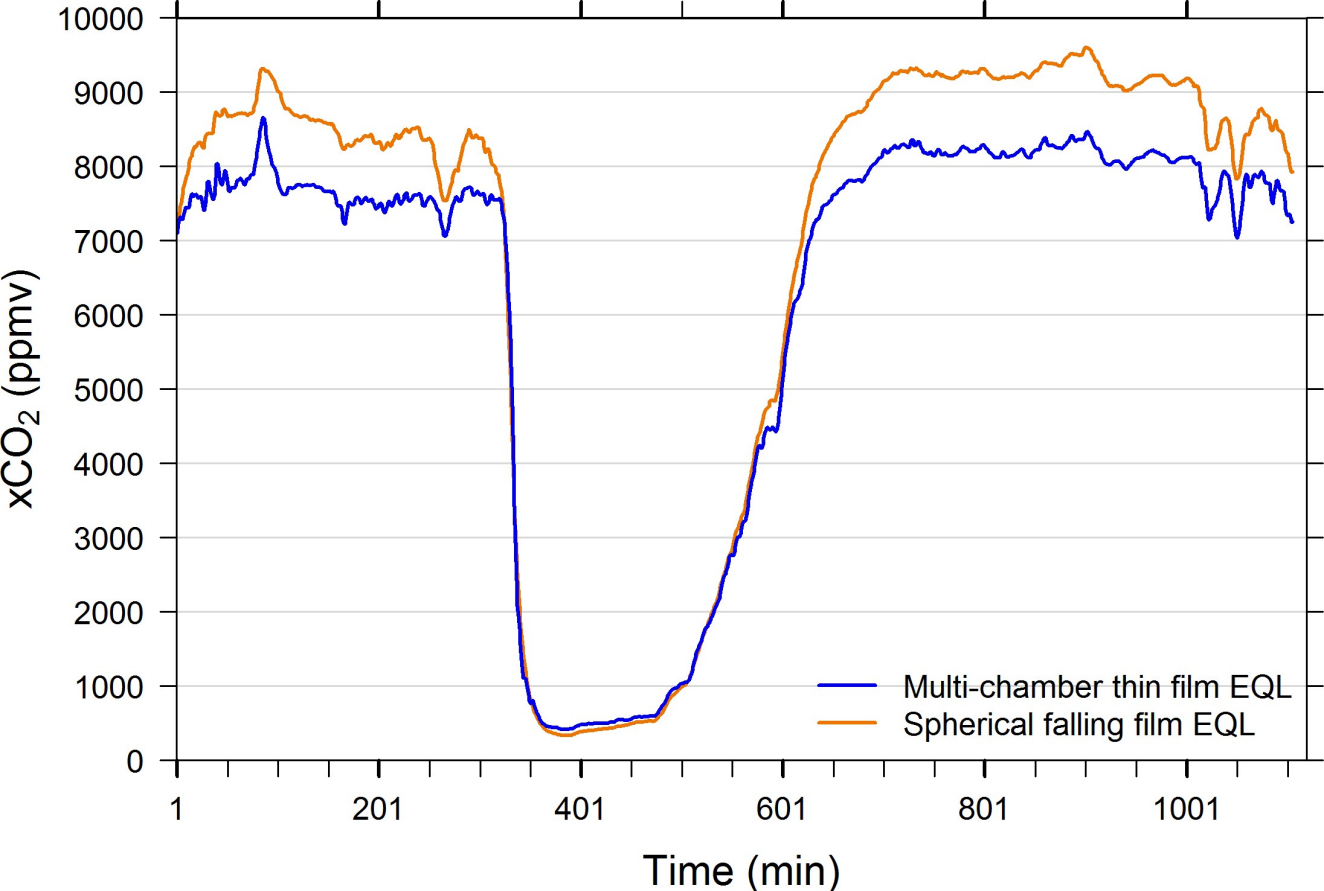
Side-by-side field measurements of multi-chamber thin film open loop and 8-in spherical falling film closed loop EQLs. Measurements were made autonomously at 1-min intervals February 27–28, 2017 in the tidal creek that drains the Kirkpatrick tidal marsh in the Rhode River, Edgewater, MD. This system is tidally driven, and CO_2_-rich water is drained during low tides. The spherical falling film EQL consistently measured substantially higher xCO_2_ values than the multi-chamber thin film EQL during low tides, and slightly lower values during high tide (mean difference ± SD = 662.7 ± 427.7 ppmv, n = 1106).

When the spherical falling film EQL was operated side-by-side with the multi-chamber thin film, open loop EQL, it was shown to have a wider working range (i.e., equilibrates to higher and lower pCO_2_ conditions). Fig 10 reveals that the water’s xCO_2_ at the SERC pier remained in the neighborhood of 200 ppmv or less over the two days of observation. The 10-in spherical falling film EQL consistently equilibrated to lower xCO_2_ values, approximately 20 ppmv lower than the multi-chamber thin film, open loop EQL (Fig 10). Likewise, under high xCO_2_ conditions (above 9,000 ppmv during low tide) the 10-in spherical falling film EQL consistently measured higher values than the multi-chamber thin film EQL (Fig 11).

**Fig 12:**
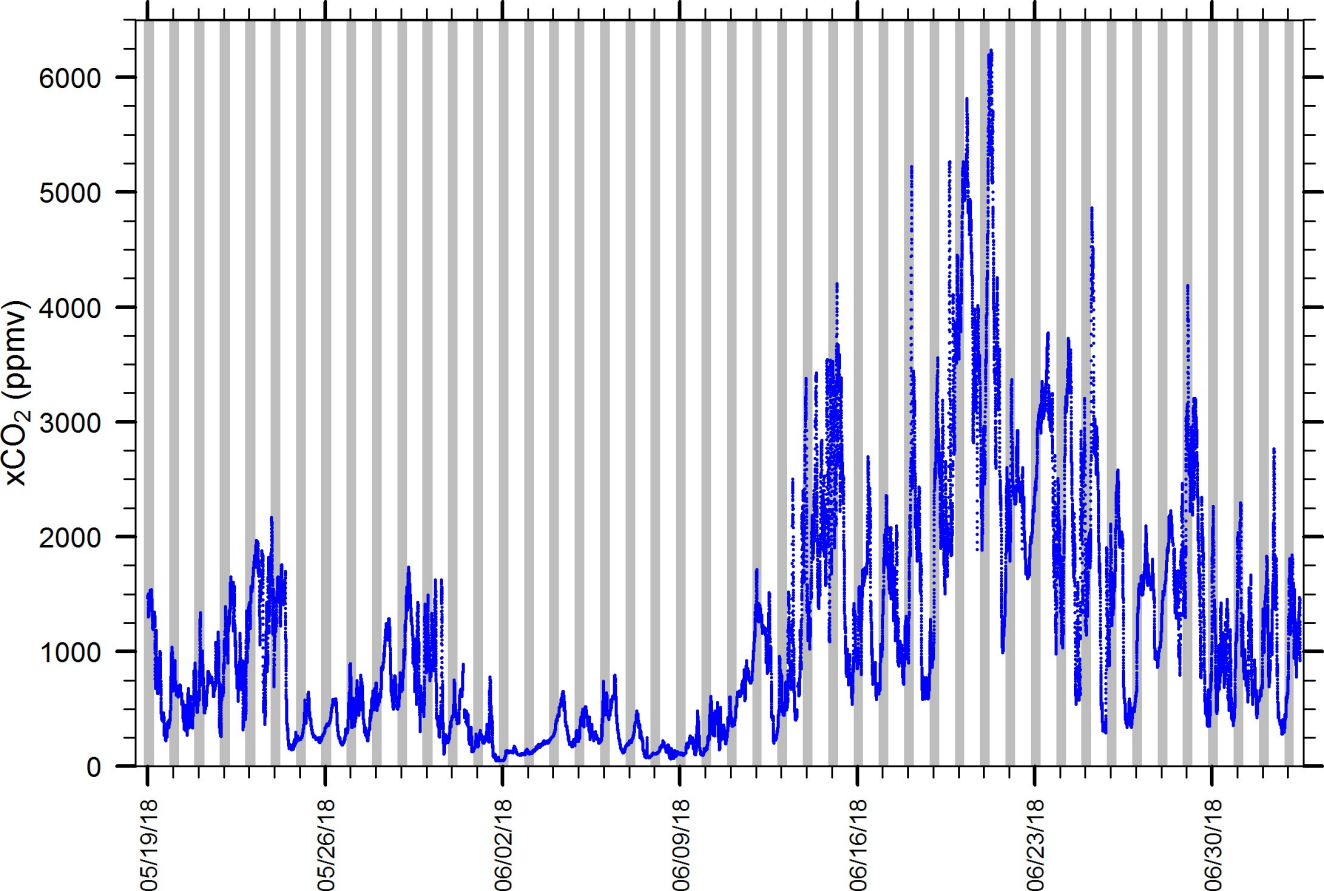
Time series (1-min intervals) of xCO_2_ from the SERC pier on the Rhode River, MD. The EQL was connected to a Senseair K30 non-dispersive infrared sensor and detects the strong biologically-driven diel cycling that is typical in warm months at this location. Gray vertical bars indicate nighttime.

We have deployed the spherical falling film EQL for months in the Chesapeake Bay with periodic cleaning (1-wk to 1-mo intervals) without failure due to clogging or freezing. Fig 12 is an 8-wk time series (1-min intervals) typical of xCO_2_ levels and dynamics observed in the Rhode River at the SERC pier during the summer (range 50–6236 ppmv, n = 65,170). During one particular 1-wk cold spell, winter air temperatures fell below freezing (min = -15°C) and the surface of Rhode River froze solid, but the spherical falling film EQL continued to operate properly without freezing. Likewise, during warm months, when the Chesapeake is exceptionally turbid and with high concentrations of phytoplankton, no EQL failures have been observed. Furthermore, during deployments aboard small motorboats, waves and rocking do not affect performance and underway transects are fully achievable.

## Discussion

Our results indicate that falling films of water over different sized spherical EQL surfaces equilibrate to the same extent. Even a small sphere (3.7-in = 9.4 cm diam) provides ample instantaneous wetted surface area (278 cm^2^) for rapid equilibration across a wide range of xCO_2_ levels and gradients. Tests using known standard CO_2_/air mixtures, confirmed equilibration to be both accurate and precise. When response times were compared with other published EQL designs, the spherical falling film EQL fell within the range of times reported for the showerhead and marble EQLs, and below the response times of the Bubble EQL (Table 3). With modest optimization efforts, such as minimizing headspace and adjustment to water and air flows, reductions in response time are feasible, in cases where more rapid equilibration may be necessary.

Falling films are typically used for industrial heat exchange processes and rely on very specific laminar flow rates to maximize efficiency and output. Although the spherical EQL design is based on a falling film model, in our case, we sought an EQL design that would provide sufficient gas exchange for rapid equilibration of trace gases, primarily CO_2_, across the diverse and variable conditions encountered during field use. The flow rates of water across the surface of this spherical EQL design produce laminar flow and generate stable and reliable surfaces for gas exchange. At a standard flow rate, if equal water velocities and thicknesses are assumed, a small sphere will have a shorter water residence time and be re-wetted more frequently than a large sphere, but the total amount of wetted surface area yielded over time will be equal. Given the convergence of gas-liquid equilibration over a wide range of sphere sizes, these assumptions appear to hold. Even if assumptions are not absolute, any differences in the wetted surface area produced per unit time, do not affect the degree of equilibration achieved. Regardless of the precise film thickness and nature of its flow, the laminar flows we have observed across a broad range of sphere sizes are stable and effectively physically capture the water to efficiently generate falling films. These films produce air-water interfaces for rapid gas exchange that are continuously renewed as the sphere’s surface is re-wetted with new water. The complex flow within the falling water continuously brings water from the sphere’s surface to air-water interface, likely further enhancing gas exchange.

In addition to free flow of turbid/debris-ridden water that resists failure due to clogging, flooding, and freezing, other advantages of the spherical falling film EQL are: 1) it is not adversely affected by tilting off vertical, such as is common when deployed on a ship, small vessel, or buoy; 2) it is easy to construct, maintain, and clean; 3) it is small enough for ease of use and has a modest energy (pumping) requirement (12 V DC, < 15 W); 4) it does not require overly exacting operational conditions (e.g., functions successfully across a broad range of water and air flow rates) to match user’s specific needs.

Based on its performance, the spherical falling film EQL provides a robust, yet simple design alternative for investigators and others seeking to equilibrate CO_2_ or other trace gases under a variety of environmental conditions and during extended deployments. Characterizing the dynamics of pCO_2_ in coastal marine systems, estuaries, and freshwaters requires extended and reliable measurements to capture the natural variability present, variability that can exceed the inter-annual variation observed in open oceans by two orders of magnitude or more on a single day (Fig 12). When paired with other sensor types, this EQL is expected to work well for individual and simultaneous measurements of assorted trace gases such as methane, radon, nitrous oxide, hydrogen sulfide, total trihalomethanes, sulfur hexafluoride, etc., that may be important in environmental and industrial monitoring. The simplicity and efficacy of the spherical falling film EQL design enable it to be paired with a wide variety of gas sensors, and depending on the application, allow a user to match it with sensors that have appropriate precision and accuracy.

## Supporting information

S1 Fig Multi-chamber thin film equilibrator. Water flows into the vertical stacks through three water intakes, washes down the inner walls creating a gas exchange surface and then drains out bottom. A water trap prevents intrusion of air through drain. Atmospheric air enters left vertical stack, passes upward, interacting with downward falling water, passes diagonally downward through external inter-stack air lines to bottom of adjacent vertical stack, repeats. Equilibrate air is dehumidified prior to entering a non-dispersive infrared gas analyzer. Measured air is exhausted to the atmosphere. In this configuration, both water and air circuits are open.
(TIFF)

S2 Fig Closed air/ closed water circuits pCO_2_ measuring apparatus used to test the accuracy of the falling film EQL against known standard gas air mixes and for measuring the response rate of a falling film EQL. Water tank volume = 5 gal. Note: a similar configuration was used to compare efficacy of EQL sphere diameters; however, water tank volume = 400 L and the tank cover was only semi-closed (i.e., closed air/ open water circuits pCO_2_ configuration). Tank included an air stone that enable enrichment by CO_2_ or stripped of CO_2_ by bubbling with atmospheric air (not shown). The 400 L water tank allowed two equilibrators to run simultaneously and for their respective equilibrated air circuits to be alternately directed to the infrared gas analyzer with a pair of 2-way valves (not shown).
(TIFF)

S3 Fig Falling film EQL (closed air circuit, see Fig 1 for detailed description) and multi-chamber thin film equilibrator EQL (open air circuit, see S1 Fig for detailed description) configurations for field measurements. Both equilibrator configurations use open water circuits (not shown), either using pumped water from the environment or from a semi-closed water tank.
(TIFF)

S1 Table Data for Fig 2. (CSV)

S2 Table Data for Fig 3. (CSV)

S3 Table Data for Fig 4. (CSV)

S4 Table Data for Fig 5. (CSV)

S5 Table Data for Fig 6. (CSV)

S6 Table Data for Fig 7. (CSV)

S7 Table Data for Fig 8. (CSV)

S8 Table Data for Fig 9. (CSV)

S9 Table Data for Fig 10. (CSV)

S10 Table Data for Fig 11. (CSV)

S11 Table Data for Fig 12. (CSV)
