# Supplementary figures and images for: A spherical falling film gas-liquid equilibrator for rapid and continuous measurements of CO2 and other trace gases
Source: PLoS One. 2019 Sep 25;14(9):e0222303. doi: 10.1371/journal.pone.0222303 (PMC6760761; doi:10.1371/journal.pone.0222303)

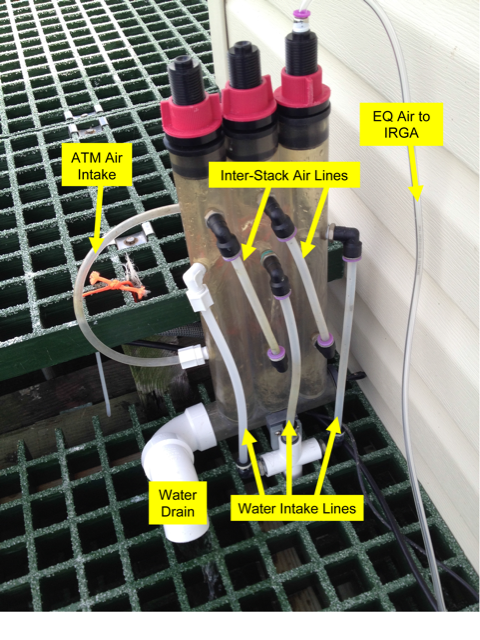

Supplement: S1 Fig — Water flows into the vertical stacks through three water intakes, washes down the inner walls creating a gas exchange surface and then drains out bottom. A water trap prevents intrusion of air through drain. Atmospheric air enters left vertical stack, passes upward, interacting with downward falling water, passes diagonally downward through external inter-stack air lines to bottom of adjacent vertical stack, repeats. Equilibrate air is dehumidified prior to entering a non-dispersive infrared gas analyzer. Measured air is exhausted to the atmosphere. In this configuration, both water and air circuits are open. (TIFF) [file pone.0222303.s001.tiff]

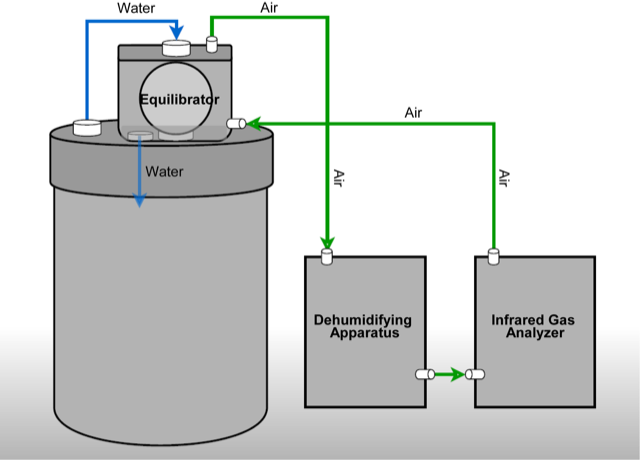

Supplement: S2 Fig — Water tank volume = 5 gal. Note: a similar configuration was used to compare efficacy of EQL sphere diameters; however, water tank volume = 400 L and the tank cover was only semi-closed (i.e., closed air/ open water circuits pCO2 configuration). Tank included an air stone that enable enrichment by CO2 or stripped of CO2 by bubbling with atmospheric air (not shown). The 400 L water tank allowed two equilibrators to run simultaneously and for their respective equilibrated air circuits to be alternately directed to the infrared gas analyzer with a pair of 2-way valves (not shown). (TIFF) [file pone.0222303.s002.tiff]

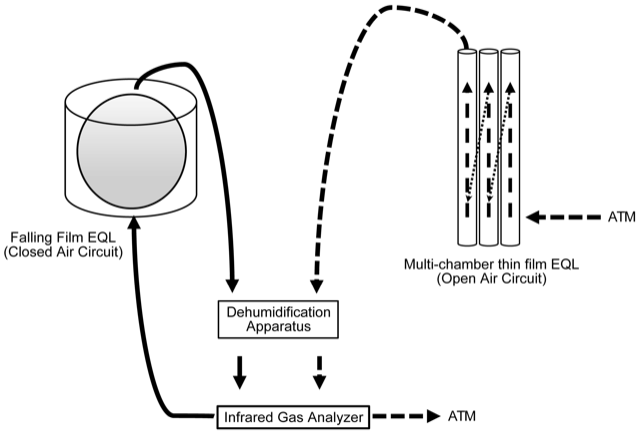

Supplement: S3 Fig — Both equilibrator configurations use open water circuits (not shown), either using pumped water from the environment or from a semi-closed water tank. (TIFF) [file pone.0222303.s003.tiff]
